# Personalized Medicine in Skull Base and Sinonasal Tumors

**DOI:** 10.3390/jpm12121983

**Published:** 2022-12-01

**Authors:** Davide Mattavelli, Paolo Bossi

**Affiliations:** 1 Unit of Otorhinolaryngology-Head and Neck Surgery, Department of Medical and Surgical Specialties, Radiological Sciences and Public Health, ASST Spedali Civili of Brescia, University of Brescia, 25123 Brescia, Italy; 2 Unit of Medical Oncology, Department of Medical and Surgical Specialties, Radiological Sciences and Public Health, ASST Spedali Civili of Brescia, University of Brescia, 25123 Brescia, Italy

Skull base and sinonasal tumors (SBSNTs) represent a considerable challenge for clinicians in view of their rarity, anatomical complexity of the site of origin, and great histological variety.

Survival rates from the 1970s to early 2000s did not significantly improve, approximately presenting a 5-year estimate of 50% [1]. During this period, the treatment strategy was based on the surgical resection of the tumor and adjuvant (chemo)radiotherapy according to risk factors, regardless of tumor histology.

In recent decades, a multidisciplinary approach and histology-driven treatment of SBSNTs has revolutionized the therapeutic management of these cancers. The evolving understanding of SBSNT biology has been paralleled by the investigation of new therapeutic regimens, such as systemic therapy, as induction chemotherapy, systemic targeted agents, and immunotherapy, even if in limited series. An analysis of the clinical data led to the assessment of the radio- and chemosensitivity of different histotypes, and the beneficial effect on the survival rates as a result of multimodality treatments became evident. Increasingly robust evidence supported the relevance of a histology-driven treatment plan, and investigations were addressed towards a precise definition of the best therapeutic strategy for specific groups of histologies. In this context, the collection of specific expertise obtained from different disciplines (i.e., pathology, radiology, ENT, neurosurgery, radiation, and medical oncology), the discussion of each clinical case in a tumor board, and the coordination among treating physicians is gaining extraordinary relevance to improve survival rates. A paradigmatic example is represented by sinonasal undifferentiated carcinoma (SNUC), an aggressive neoplasm historically burdened by an ominous prognosis. The introduction of multimodality treatment relevantly improved prognosis to close to 80% at 5 years. Induction chemotherapy was introduced as a prognostic biomarker and treatment modifier to select candidates for radio(chemo)therapy (responders) vs. surgery and adjuvant treatments (non-responders) [2].

Simultaneously, relevant innovations in surgery and radiotherapy were introduced, including the endoscopic endonasal resection of sinonasal cancers, expanded endoscopic approaches to the skull base, and intensity-modulated and heavy-ion radiation therapies. In general, these approaches guaranteed equal (or superior) chances of local control, but remarkably reduced the associated morbidity.

The above-mentioned novelties considerably improved the efficacy and quality of treatments in the last two decades. However, a relevant proportion of patients still have poor prognoses despite the intensified regimen of treatments, which seem to have reached a plateau in their anti-tumor activity. In this subset of patients, tumor biology largely drives the outcome. Future evolutions in SBSCTs are likely to rely on refinements of multimodality protocols and an improved profiling of tumor biology. Multi-omics analysis has the potential to greatly improve our understanding of these diseases and provide us with biomarkers to better characterize these tumors on multiple levels, i.e., the diagnosis, prognostic stratification, and prediction of response to treatment.

Within the framework of histology-driven treatment, pathological diagnosis is of utmost importance to create a correct therapeutic plan. Owing to the rarity and histological heterogeneity of these tumors, a change in diagnosis after a second revision is frequent (up to 24% in specific histotypes) and represents a negative prognostic factor [3]. The research conducted in pathology may contribute to the personalized and multidisciplinary treatment of SBSNTs at multiple levels. First, the definition of pathological entities through objective (molecular and/or immunohistochemical) biomarkers can reduce misdiagnosis rates and improve pathological classifications. Of note, these biomarkers may also convey prognostic information and allow for the identification of a subset of tumors with specific biological aggressiveness. Therefore, the marker may have a mostly diagnostic relevance (i.e., IDH-2 mutation for SNUC), lead to the definition of a new pathological entity (i.e., INI-1-deficient carcinoma as a segregation of SNUC), or open the possibility for new, tailored, targeted approaches (i.e., HER2 amplification or EGFR mutation). Multiomics molecular analysis (genomics, transcriptomics, and methylomics) in poorly differentiated sinonasal cancers is shedding light on these groups of histologies, paving the way towards molecular-based tumor classifications [4,5] that can translate into precision-medicine therapeutics.

Radiology has played a pivotal role in diagnosis by improving the definition of tumor local extension and early tumor relapse thanks to the evolution of magnetic resonance imaging. Radiomics is a novel, promising field of investigation that aims to extract quantitative textural information from medical imaging thanks to complex mathematical analyses and artificial intelligence algorithms. Theoretically, it can provide a considerable amount of objective information (diagnostic, prognostic, and predictive) that cannot be retrieved or analyzed by the human eye or brain.

Recent experiences have shown its promising role in the anticipation of tumor response to treatment. In an Italian series, radiomics signatures predicted the response to induction chemotherapy better than classical RECIST and volumetric criteria [6]. The identification of responders at pre-treatment imaging could bypass the use of induction chemotherapy as a treatment modifier and avoid unnecessary toxicity in non-responders.

Molecular analyses are probably the most promising field of investigation in tumor biological profiling. Genomics and the tumor mutational landscape has been extensively studied in the literature. Preliminary evidence has demonstrated the possible role of genomic signatures in the prediction of responses to induction chemotherapy [7] and prognostic stratification [8]. Gene expression profiling can convey even more useful information on the status of different genes, and better describe the biological behavior of a tumor in terms of local invasiveness or metastases. A promising means of investigation is the integration of multiple molecular analysis in a single study framework. A paradigm of this approach is the work of Classe et al. on esthesioneuroblastoma (ENB). The authors conducted a comprehensive molecular analysis, including genomics, transcriptomics, methylomics, and proteomics, which allowed for the definition of two distinct entities: neural ENB (well-differentiated and indolent lesions associated with longer survival rates) and basal ENB (poorly differentiated and aggressive tumors with ominous survival rates) [9]. Interestingly, a transcriptomic analysis obtained from another series identified six pathways with prognostic significance and indirectly confirmed the dual characterization (neural vs. basal) in ENB [10].

Molecular studies can also identify therapeutic targets to inspire new pharmaceutical options. At present, several therapeutic targets (mutations and/or altered gene expression pathways) are potentially druggable; however, preliminary clinical data are disappointing, which suggests that the biological profile of these tumors is very complex and far from being fully elucidated. Nevertheless, the thorough definition of tumor molecular alterations is a prerequisite of any targeted therapy; therefore, investigations in this area are needed to improve survival rates in patients who are not curable with conventional therapies.

Finally, the tumor–host interplay and immune contexture has been largely studied in the last decade in many cancer types, including head and neck tumors. Due to their rarity, evidence in SBSNT is still sparse. This represents a new frontier to be explored, and would represent an essential prerequisite to include immunotherapy in the therapeutic armamentarium for SBSNTs. To our knowledge, the first phase-II study has been initiated to investigate immunotherapy in the treatment of sinonasal, poorly differentiated carcinomas, by employing the combination of pembrolizumab, docetaxel, and platinum in patients with stages II–IVb squamous cell carcinomas of the nasal cavity/paranasal sinuses (NCT05027633).

In conclusion, treatment strategies used for SBSNTs have greatly improved in recent decades, and these patients can now benefit from a greater chance of being cured with reduced toxicity outcomes. Nevertheless, a non-negligible fraction of patients still presents a poor outcome despite the intensified, histology-driven treatments. Historical weapons, such as surgery and radiotherapy, have undergone a great evolution in the last three decades, which has led to a relevant increase in the rate of tumor local control and a simultaneous decrease in morbidity rates. However, this evolution apparently came to a plateau, and further improvements in these fields are unlikely to significantly improve survival rate when considered on their own.

On the other hand, multi-omics analyses are the best candidates to achieve a thorough biological profiling of SBSNTs, which, in turn, should lead to an overall improvement in the management of these diseases. From a therapeutical standpoint, molecular analysis can provide the following relevant insights at multiple levels: (a) define targets for new therapeutic agents; (b) describe biomarkers to predict treatment response and/or stratify prognosis; and (c) better depict tumor behavior and chemo-radiosensitivity in a single patient and help in the planning of multidisciplinary regimens. Overall, the final goal is the achievement of a greater personalization of the therapeutic strategy, with the early identification of “bad” tumors where treatment should be intensified, and “good” ones in which deintensification can possibly guarantee equal survival with fewer toxicity outcomes.
